# Supplementary material for: Longitudinal quantification of Bifidobacterium longum subsp. infantis reveals late colonization in the infant gut independent of maternal milk HMO composition
Source: Nat Commun. 2024 Jan 30;15:894. doi: 10.1038/s41467-024-45209-y (PMC10827747; doi:10.1038/s41467-024-45209-y)
Supplement: Supplementary file 1 — Supplementary Information [file 41467_2024_45209_MOESM1_ESM.pdf]

Supplementary figure 1

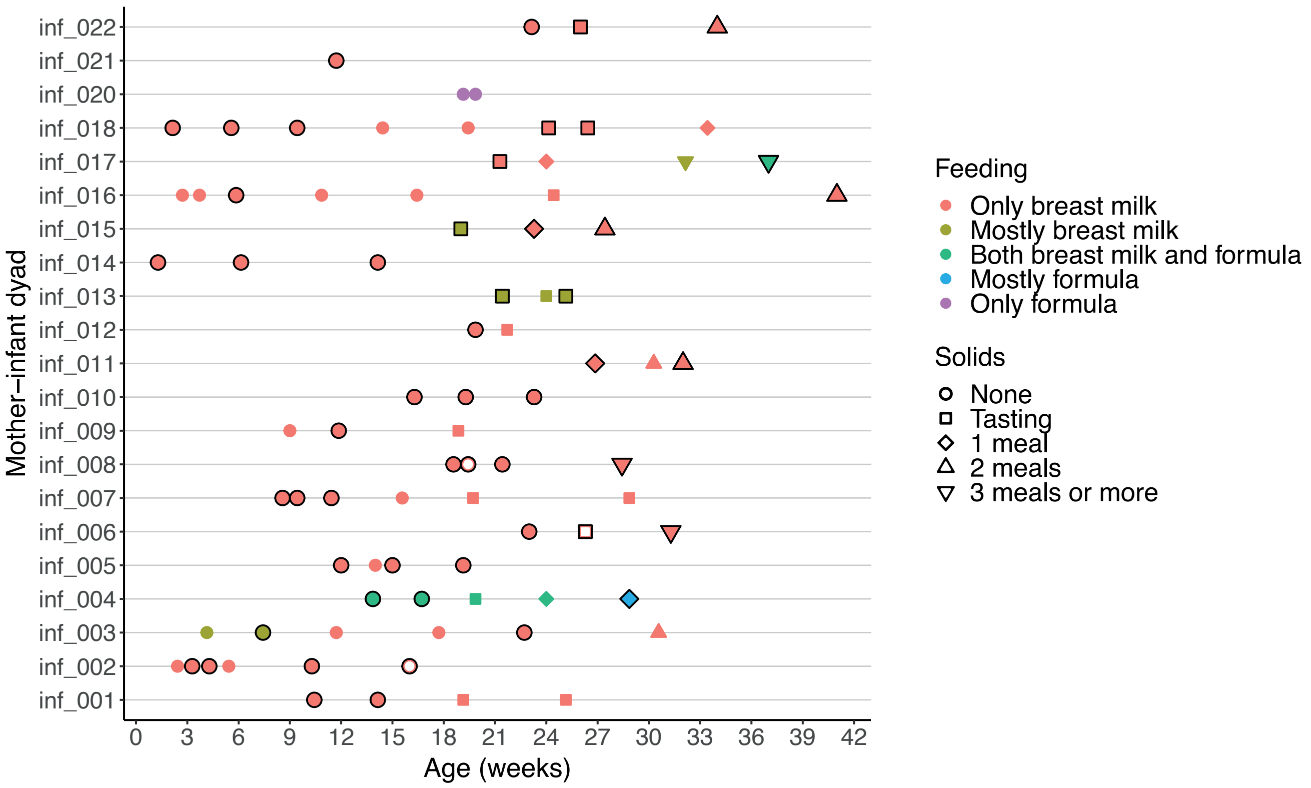

Supplementary Figure 1: Cohort Overview. Overview of all samples collected as part of the cohort. Each data point represents a pair of an infant stool sample and a mother's breast milk sample. Data points with a black border indicate breast milk samples used for HMO quantification, while filled-in data points represent stool samples subjected to metagenomic sequencing. The color of the points represents the feeding practice, and the shape of the points indicates the amount of solids consumed by the infant per day.

Supplementary figure 2

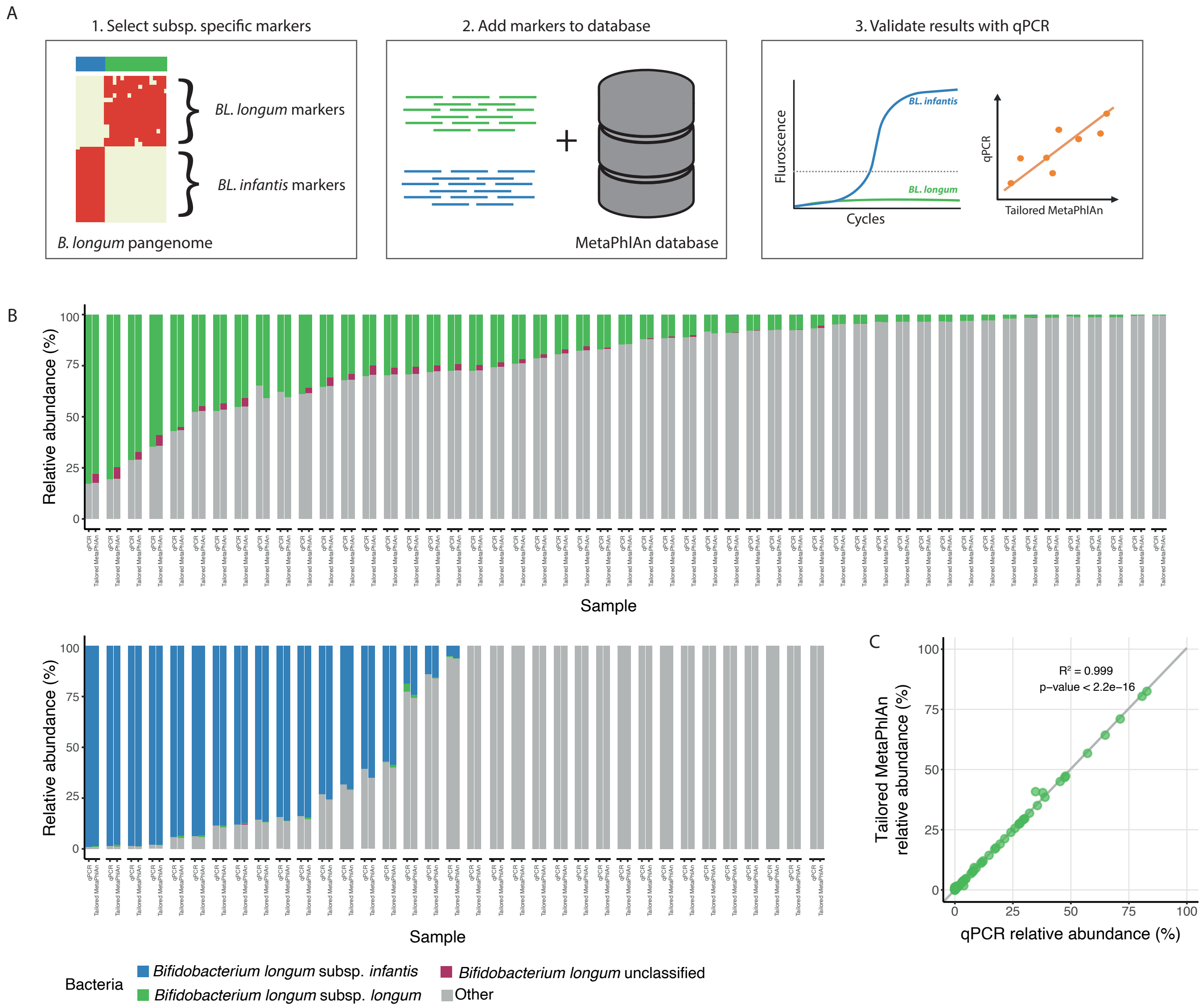

Supplementary Figure 2: *B. longum* subspecies specific marker genes. (A) Illustration depicting the process of selecting and validating *B. longum* subspecies marker genes and their addition to the MetaPhlAn database. **(B)** Relative abundance of *B. longum* subspecies using qPCR and the customized MetaPhlAn method. Each sample is represented by two bars, one for each method. *B. longum* that was not assigned to either subspecies by our tailored MetaPhlAn method was classified as *B. longum* unclassified (burgundy). **(C)** Validation of the computational approach, comparing the tailored MetaPhlAn marker-gene quantification results to the experimental qPCR results for *BL. longum*. The quantification using MetaPhlAn includes bacteria assigned as *B. longum* unclassified.

Supplementary figure 3

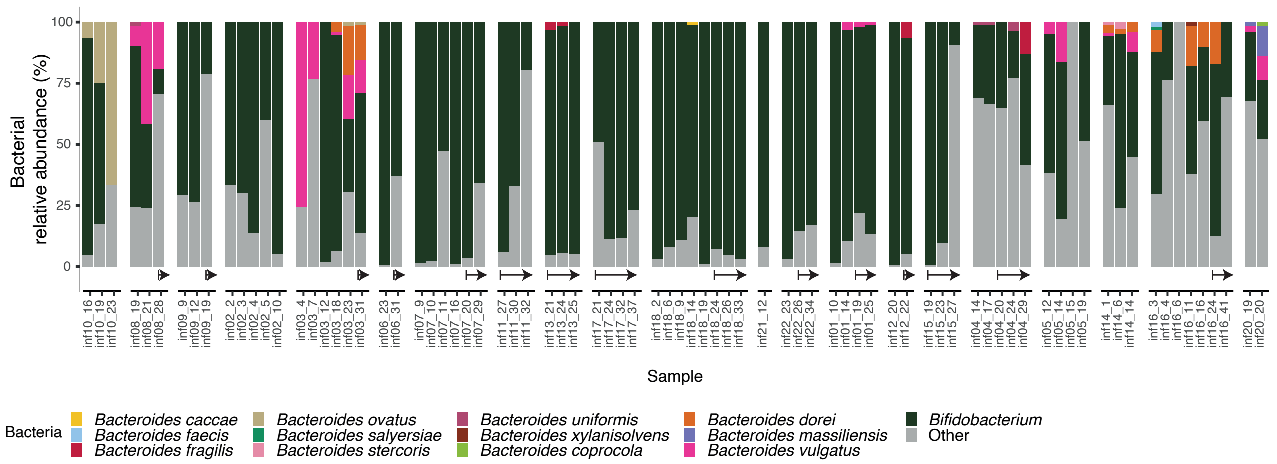

Supplementary Figure 3: Bacterial composition of the infant gut. Microbial composition in all samples highlighting the *Bifidobacterium* genus and *Bacteroides* species. All other bacteria are classified as "other" (gray). Arrows indicate samples taken after the introduction of solid food.

Supplementary figure 4

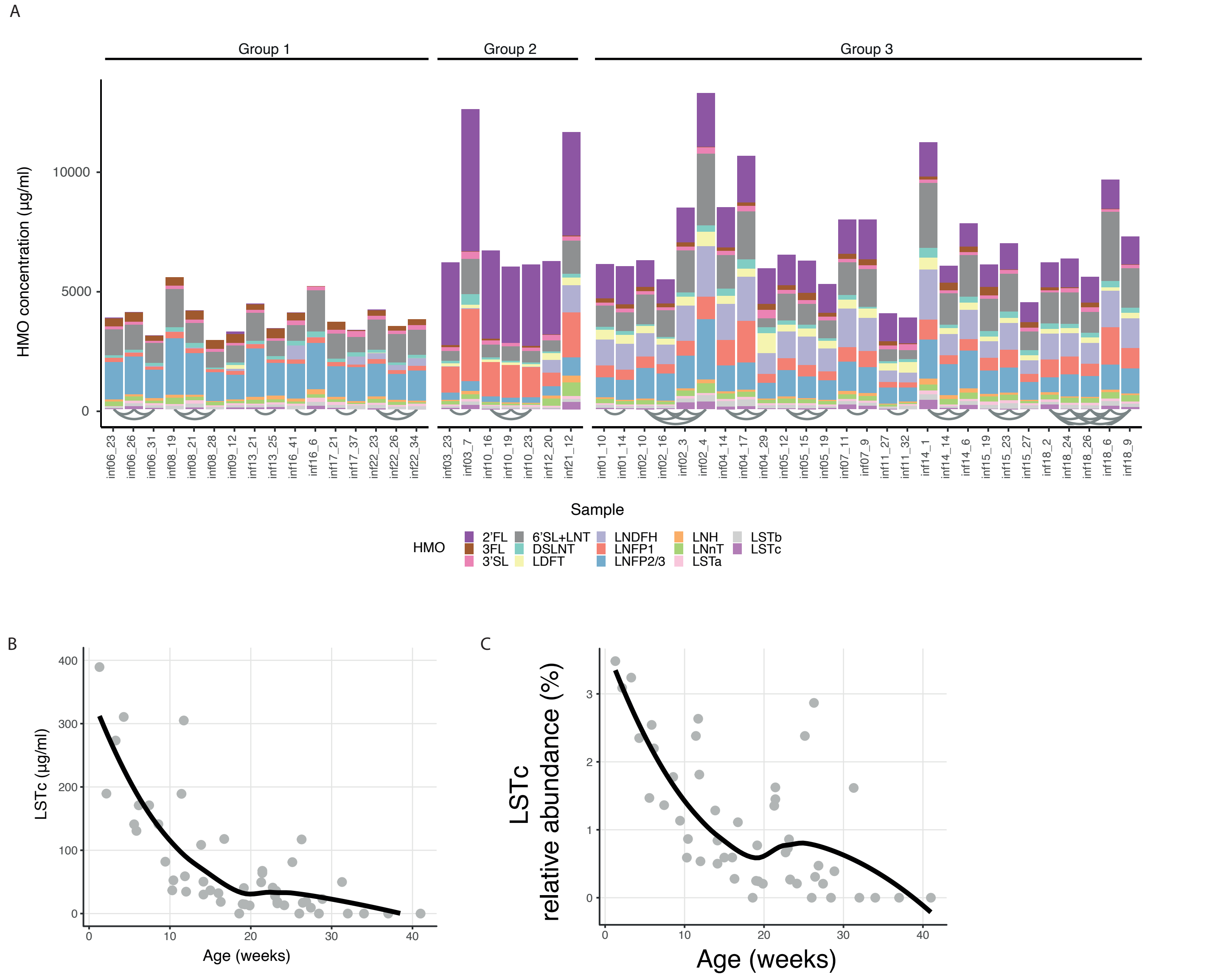

Supplementary Figure 4: HMO composition in mothers' milk. (A) Absolute concentration of 16 HMOs measured in mothers' milk. Samples are categorized into three groups based on their HMO profiles, and arches connect samples obtained from the same mother. **(B-C)** LSTc **(B)** absolute concentration and **(C)** relative abundance over time in all breast milk samples measured. The black line represents the pattern.

Supplementary figure 5

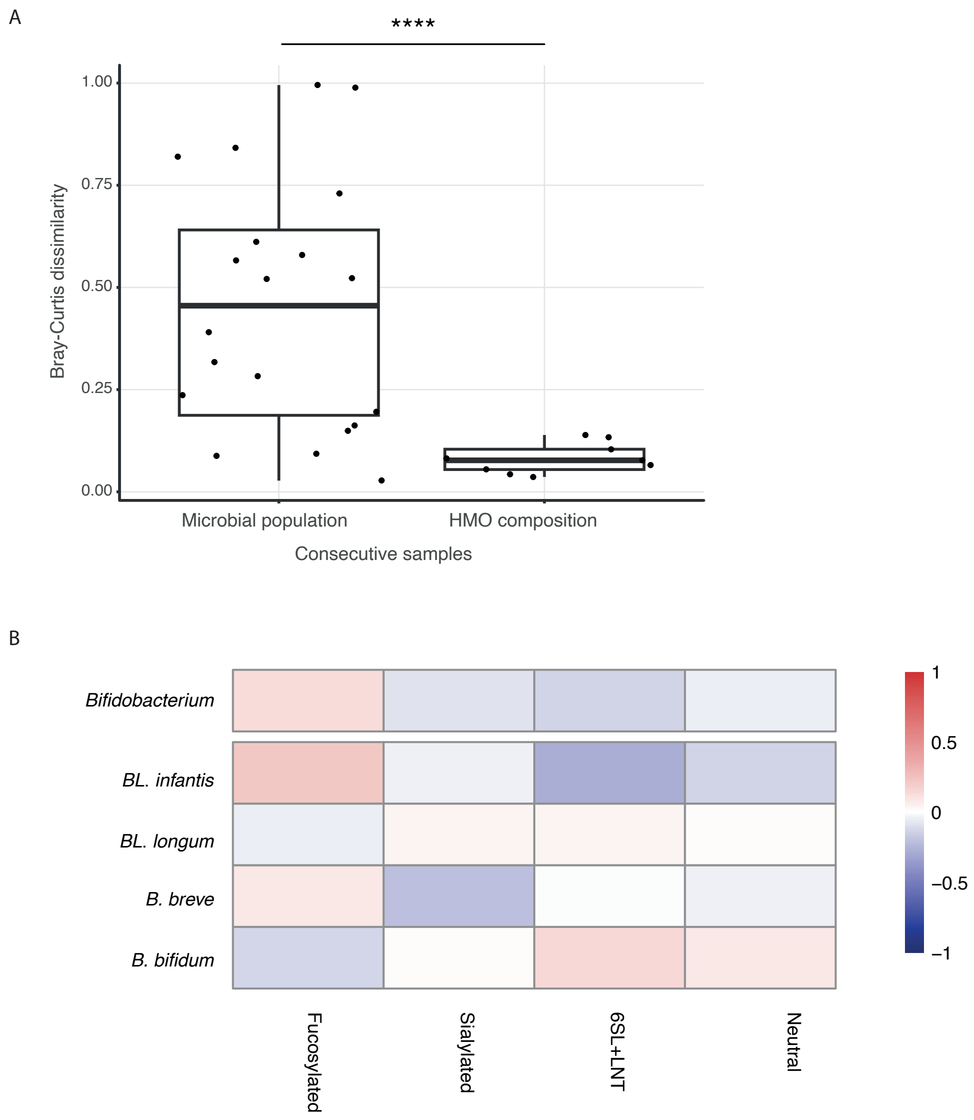

Supplementary Figure 5: HMO composition in mothers' milk and the infant gut *Bifidobacterium* population. (A) Bray-Curtis dissimilarity of the microbial population in consecutive infant gut samples compared to the Bray-Curtis dissimilarity of HMO composition in consecutive mother milk samples (two tailed t-test, ****: p ≤0.0001) . **(B)** Pearson correlations between HMO types (neutral, fucosylated and sialylated) and the *Bifidobacterium* genus, as well as the main individual species and subspecies (*BL. infantis*, *BL. longum*, *B. breve* and *B. bifidum*). None of these correlations were found to be statistically significant.

Supplementary Figure 6

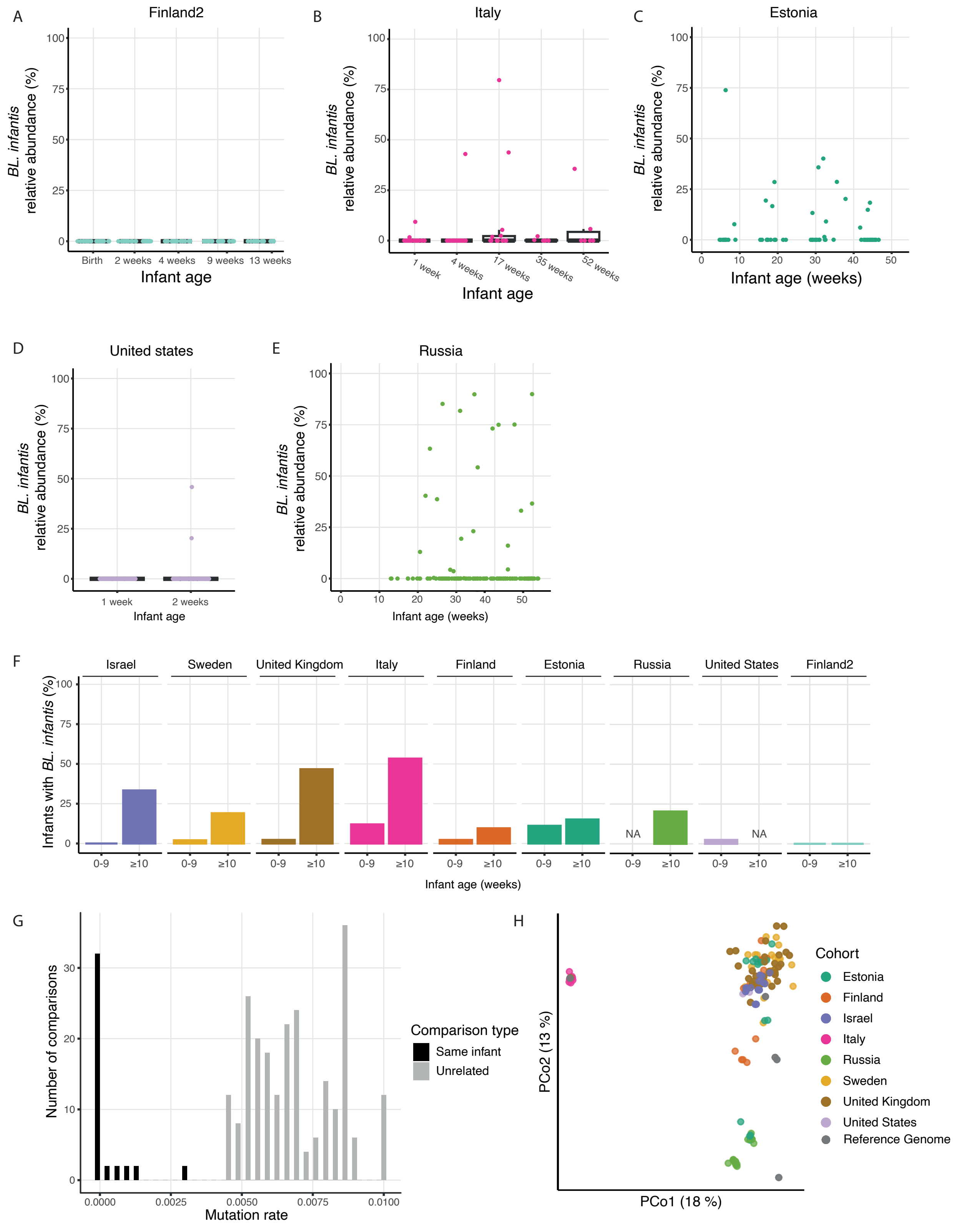

Supplementary Figure 6: *BL. infantis* colonization in the infant gut in multiple geographical locations. (A-E) Relative abundance of *BL. infantis* at different time points in samples from **(A)** Finland **(B)** Italy **(C)** Estonia **(D)** United states and **(E)** Russia. **(F)** Percentage of infants harboring *BL. infantis* at any time point, divided to the first 10 weeks of life and starting at 10 weeks and on in all cohorts. NA represented groups with no available data. **(G)** Histogram of mutation rates in *BL. infantis* marker-genes within the same infant (black) and between unrelated infants (gray) in our Israeli cohort. **(H)** Principal component analysis revealing the SNP rates of *BL. infantis* marker-genes in all samples colored based on geographic location.

Supplementary Figure 7

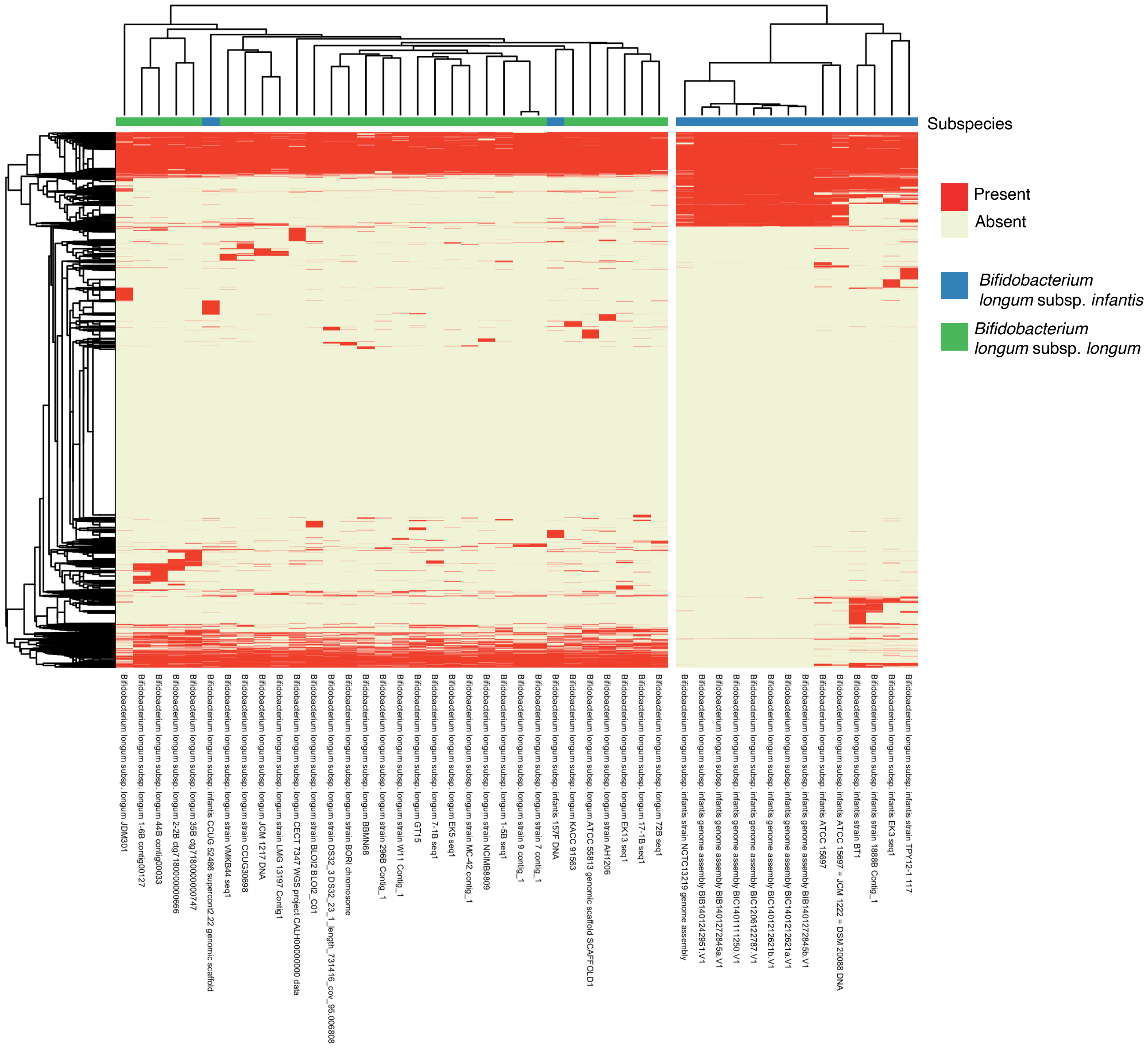

Supplementary Figure 7: Pangenome of all *BL. infantis* and *BL. longum* reference genomes. Presence or absence of all genes in the *B. longum* pangenome in all reference genomes used in the study. Reference genomes are marked based on their annotation: *BL. infantis* (blue) or *BL. longum* (green). The reference genomes are clustered into two main groups, highlighting the mis-annotation of two *BL. infantis* reference genomes.
