# Supplementary material for: Longitudinal quantification of Bifidobacterium longum subsp. infantis reveals late colonization in the infant gut independent of maternal milk HMO composition
Source: Nat Commun. 2024 Jan 30;15:894. doi: 10.1038/s41467-024-45209-y (PMC10827747; doi:10.1038/s41467-024-45209-y)
Supplement: Supplementary file 8 — Reporting Summary [file 41467_2024_45209_MOESM8_ESM.pdf]

nature portfolio

Corresponding author(s): Moran Yassour

Last updated by author(s): Nov 20, 2023

Reporting Summary

Nature Portfolio wishes to improve the reproducibility of the work that we publish. This form provides structure for consistency and transparency in reporting. For further information on Nature Portfolio policies, see our Editorial Policies and the Editorial Policy Checklist.

Statistics

For all statistical analyses, confirm that the following items are present in the figure legend, table legend, main text, or Methods section.

n/a	Confirmed	
☐	☒	The exact sample size (*n*) for each experimental group/condition, given as a discrete number and unit of measurement
☒	☐	A statement on whether measurements were taken from distinct samples or whether the same sample was measured repeatedly
☐	☒	The statistical test(s) used AND whether they are one- or two-sided<br>*Only common tests should be described solely by name; describe more complex techniques in the Methods section.*
☐	☒	A description of all covariates tested
☐	☒	A description of any assumptions or corrections, such as tests of normality and adjustment for multiple comparisons
☐	☒	A full description of the statistical parameters including central tendency (e.g. means) or other basic estimates (e.g. regression coefficient) AND variation (e.g. standard deviation) or associated estimates of uncertainty (e.g. confidence intervals)
☐	☒	For null hypothesis testing, the test statistic (e.g. *F*, *t*, *r*) with confidence intervals, effect sizes, degrees of freedom and *P* value noted<br>*Give P values as exact values whenever suitable.*
☒	☐	For Bayesian analysis, information on the choice of priors and Markov chain Monte Carlo settings
☒	☐	For hierarchical and complex designs, identification of the appropriate level for tests and full reporting of outcomes
☐	☒	Estimates of effect sizes (e.g. Cohen's *d*, Pearson's *r*), indicating how they were calculated

Our web collection on statistics for biologists contains articles on many of the points above.

Software and code

Policy information about availability of computer code

Data collection	Metagenomic reads from stool samples were obtained using Illumina single-end 150bp sequencing on a NextSeq 500 device.<br>HMO concentration in breast milk was quantified high perforusing mance liquid chromatography with fluorescence detection (HPLC-FLD)
Data analysis	Our tailored MetaPhlAn database is available on our GitHub page (https://github.com/yassourlab/MetaPhlAn-B.infantis/).<br>Data analysis was performed using PanPhlan3, Blastn 2.12.0, MetaPhlAn 4, StrainPhlAn 4, HUMAnN 3, Bowtie2 (2.4.5-1,) fastq-mcf (ea-utils, 1.05).<br>In addition, we used an in-house R script utilizng the fallowing packages:<br>dplyr(1.1.2), tidyr(1.3.0), tidyverse (2.0.0), ggplot2(3.4.2), ggforce(0.4.1), RColorBrewer(1.1-3), pals(1.7), pheatmap, vegan(2.6-4), ape (5.7-1), ggtree (3.6.2) and ggsankey (0.0.99999).

For manuscripts utilizing custom algorithms or software that are central to the research but not yet described in published literature, software must be made available to editors and reviewers. We strongly encourage code deposition in a community repository (e.g. GitHub). See the Nature Portfolio guidelines for submitting code & software for further information.

Data

Policy information about availability of data

All manuscripts must include a data availability statement. This statement should provide the following information, where applicable:

- Accession codes, unique identifiers, or web links for publicly available datasets
- A description of any restrictions on data availability
- For clinical datasets or third party data, please ensure that the statement adheres to our policy

Human-filtered metagenomic sequencing data was deposited in SRA under BioProject PRJNA994433.

Our tailored MetaPhlAn database is available on our GitHub page and is mentioned in the manuscript under code availability .

Research involving human participants, their data, or biological material

Policy information about studies with human participants or human data. See also policy information about sex, gender (identity/presentation), and sexual orientation and race, ethnicity and racism.

Reporting on sex and gender	No relevant analysis was performed
Reporting on race, ethnicity, or other socially relevant groupings	No relevant analysis was performed
Population characteristics	Healthy infants and their mothers were recruited between the age of 2 weeks and 1 year. aLL infants and mothers lived in Israel and were healthy throughout the study.
Recruitment	Study was publicized over social media and through word of mouth to mothers in the area of Jerusalem, Israel.<br>As a result there may be a bias to mothers we knew personally and those that lived in the area of the university and therefore were easy to recruit and to collect samples from.
Ethics oversight	All mothers have agreed to participate in our study, which was approved by the Hebrew University's Institutional Review Board (IRB, approval number 20042021), and signed our consent forms.

Note that full information on the approval of the study protocol must also be provided in the manuscript.

Field-specific reporting

Please select the one below that is the best fit for your research. If you are not sure, read the appropriate sections before making your selection.

[x] Life sciences [ ] Behavioural & social sciences [ ] Ecological, evolutionary & environmental sciences

For a reference copy of the document with all sections, see nature.com/documents/nr-reporting-summary-flat.pdf

Life sciences study design

All studies must disclose on these points even when the disclosure is negative.

Sample size	21 mother- infants dyads with 80 infant stool samples and 50 breast milk samples were collected.<br>We stopped recruitment after 6 months of collecting, after checking we had diverse sampling across ages and diverse microbiome profiles.
Data exclusions	When comparing breast milk and stool samples, only stool sampled that had a matching breast milk sample were included.<br>In addition, when looking at infants to secreter and non-secreter mothers (Figure 4B), one infant which was not breastfed (inf20) was excluded, due to irrelevance.
Replication	This is a descriptional study with no experiments therefore no replications were performed.
Randomization	None of are analyses required randomization
Blinding	All analysis was performed without knowing which samples came from which subject

Reporting for specific materials, systems and methods

We require information from authors about some types of materials, experimental systems and methods used in many studies. Here, indicate whether each material, system or method listed is relevant to your study. If you are not sure if a list item applies to your research, read the appropriate section before selecting a response.

Materials & experimental systems

n/a	Involved in the study
☒	☐ Antibodies
☒	☐ Eukaryotic cell lines
☒	☐ Palaeontology and archaeology
☒	☐ Animals and other organisms
☒	☐ Clinical data
☒	☐ Dual use research of concern
☒	☐ Plants

Methods

n/a	Involved in the study
☒	☐ ChIP-seq
☒	☐ Flow cytometry
☒	☐ MRI-based neuroimaging
