# Supplementary material for: Longitudinal quantification of Bifidobacterium longum subsp. infantis reveals late colonization in the infant gut independent of maternal milk HMO composition
Source: Nat Commun. 2024 Jan 30;15:894. doi: 10.1038/s41467-024-45209-y (PMC10827747; doi:10.1038/s41467-024-45209-y)
Supplement: Supplementary file 9 — Description of additional supplementary files [file 41467_2024_45209_MOESM9_ESM.pdf]

Supplementary Data 1: Metadata of all samples and infants that participated in this study.

Supplementary Data 2: *Bifidobacterium longum* reference genomes. Includes the annotation for each genome and whether it was used in our study to find subspecies specific marker-genes.

Supplementary Data 3: Marker genes added to MetaPhlAn database in order to quantify *BL. infantis* and *BL. longum.*

Supplementary Data 4: Quantification of 16 HMOs in breast milk samples (ug/ml).

Supplementary Data 5: MetaPhlAn results of the relative abundance of all bacteria at species level in all infant gut samples
